# Supplementary material for: Coarse-grained molecular dynamics-guided immunoinformatics to explain the binder and non-binder classification of Cytotoxic T-cell epitope for SARS-CoV-2 peptide-based vaccine discovery
Source: PLoS One. 2023 Oct 5;18(10):e0292156. doi: 10.1371/journal.pone.0292156 (PMC10553366; doi:10.1371/journal.pone.0292156)
Supplement: Attachment — Submitted filename: Response to Reviewers.pdf [file pone.0292156.s001.pdf]

Dear Prof.,

We would like to thank you for having your time to review our manuscript. Your critical and positive suggestions really help us to improve the manuscript substantially. We hope that the current state of the manuscript addresses all of your concerns and fulfill the requirement to be accepted in PLOS One. Please find our point-by-point responses to your comments. Thank you very much.

<table>
<tr><th>Reviewer’s Comment</th><th>Author’s Reply</th></tr>
<tr><td>It is true that the choice of T-cell-based epitopes may confer long-term protection against infection. However, short-term protection can't be neglected, and it is still the most viable way to ensure vaccine efficacy in clinical setting. B-cell response may be weak, but it is much easier to measure with simple biomedical lab setting. Determining vaccine efficacy from T-cell titers will possibly need NGS machine, and not all hospital and clinics have that.

You need to narrate a stronger argument both in discussion and intro, why should the B-cell epitopes be neglected here.

Because all existing vaccine in the market, whether it is live attenuated, mRNA, recombinant, and others, worked by inducing the B-cell response and they have been proven to slow down, if not to stop, the progression of COVID-19 pandemics.

Although it is not your forte, you need to think or predict how to measure the vaccine efficacy later.</td><td>Thank you so much for your kind suggestions.

Our research is not meant to disregard the significance of B-cell epitopes in the context of vaccine development. Instead, we choose the T-cell epitopes discovery as a case study to incorporate the molecular detail of peptide-MHC binding. We agree that ideal vaccines should elicit both B-cell (neutralizing antibody) and T-cell responses, including CD8+ and CD4+ T-cell responses. However, in Ad26.COV2.S and BNT162b2 case studies, T-cell responses have been found to have cross-reactivity against different variants of SARS-CoV-2, including the Omicron variant, but not humoral response [1]. CD8+ T cells have been shown to contribute to protection against SARS-CoV-2, especially when antibody responses are suboptimal. Therefore, the inclusion of T cell responses in addition to antibody responses in vaccines is important for robust protection against severe disease caused by SARS-CoV-2. These statements have been added in the Introduction section (line number 31-38):

“This study is not meant to disregard the significance of B-cell epitopes in the context of vaccine development. Rather, we choose the T-cell epitopes discovery as a case study to incorporate the molecular detail of peptide-MHC binding. Ideally, vaccines should elicit B-cell (neutralizing antibody) and T cell responses, including CD8+ and CD4+ T cell responses. However, in Ad26.COV2.S and BNT162b2 case studies, T cell responses have been found to have cross-reactivity against different variants of SARS-CoV-2, including the Omicron variant, but not humoral response [1]. CD8+ T cells have been shown to contribute to protection against SARS-CoV-2, especially when antibody responses are suboptimal. Therefore, the inclusion of T cell responses in addition to antibody responses in vaccines is important for robust protection against severe disease caused by SARS-CoV-2”

Regarding the immune response elicited by mRNA vaccine, it can elicit both B-cell and T-cell responses, including CD4+ and CD8+ T-cell responses (as reviewed in [2]).

About the measurement method of T-cell response titer, several ways can be used. One method is the enzyme-linked immunospot (ELISPOT) assay, which measures the number of cytokine-producing T cells in response to a specific antigen [3]. Another method is flow cytometry,</td></tr>
</table>

	which can be used to quantify the number of T cells expressing specific surface markers or intracellular cytokines [4]. Additionally, the enzyme-linked immunosorbent assay (ELISA) can be used to measure the concentration of specific cytokines or other molecules produced by T cells [5]. These methods provide quantitative measurements of T cell response titer and can be used to assess the magnitude and quality of T-cell responses in various contexts, such as infectious diseases, autoimmune disorders, and cancer immunotherapy. Moreover, the interferon test, commonly known as IGRA, has been widely used to measure T-cell responses for latent tuberculosis infection [6,7], and Covid-19 diagnostic test [8,9]. These explanations have been added in the Discussion section (line number 448-457):<br><br>"*Furthermore, there are several methods to measure the vaccine efficacy in terms of T cell response. One method is the enzyme-linked immunospot (ELISPOT) assay, which measures the number of cytokine-producing T cells in response to a specific antigen [3]. Another method is flow cytometry, which can be used to quantify the number of T cells expressing specific surface markers or intracellular cytokines [4]. Additionally, the enzyme-linked immunosorbent assay (ELISA) can be used to measure the concentration of specific cytokines or other molecules produced by T cells [5]. These methods provide quantitative measurements of T cell response titre and can be used to assess the magnitude and quality of T cell responses in various contexts, such as infectious diseases, autoimmune disorders, and cancer immunotherapy. Moreover, the interferon test, commonly known as IGRA has been widely used to measure T-cell responses for latent tuberculosis infection* [6,7], *and Covid-19 diagnostic test* [8,9]*.*"
What is the gap between the available vaccine design with yours? It seemed that there are already a lot of COVID-19 epitopes-based vaccine design, and some of them combining both T-cell and B-cell epitopes. So this kind of design could potentially confer both long and short-term protections, and could be measured with its antibody titers only. Please kindly check this link in PUBMED, and kindly justify the available gap!<br>https://pubmed.ncbi.nlm.nih.gov/?term=T-cell+epitope+sars-cov-2+vaccine	This paper aims to enhance epitope-based vaccine development by employing molecular dynamics simulations to distinguish between true positive and false positive binder peptides. It is for the first time that immunoinformatics screening incorporates the molecular binding mechanisms of peptide-MHC interactions to validate binding affinities predicted by sequence-based predictor programs. These statements have been added to the Introduction section (line number 68-71):<br><br>"*This paper aims to enhance epitope-based vaccine development by employing molecular dynamics simulations to distinguish between true positive and false positive binder peptides. It is for the first time that immunoinformatics screening incorporates the molecular binding mechanisms of peptide-MHC interactions to validate binding affinities predicted by sequence-based predictor programs.*"<br><br>Our findings enable vaccine designers to utilize molecular dynamics simulations for definitive binding category classification. The established concept from this study holds the potential for subsequent refinement, thereby augmenting the success rate of authentic epitope

	discovery in vaccine development. These statements have been added to the Discussion section (line number 457-460):<br><br>*“Our findings enable vaccine designers to utilize molecular dynamics simulations for definitive binding category classification. The established concept from this study holds the potential for subsequent refinement, thereby augmenting the success rate of authentic epitope discovery in vaccine development*.”
Pertaining your methodology, did you incorporate the latest finding of omicron subvariant (XBB)? If you don't, you need to declare this condition as limitation of your study.	At that time, the sequence of the XBB variant was unavailable. Therefore, the conservation analysis encompassed other variants while excluding XBB. This limitation has been added to the Discussion section (line number 188-189):<br><br>“*Nevertheless, this study was limited to include Wild-Type Omicron, and Delta variants. The recent XBB variant has not been added to the conservancy analysis”.*
Only after reading thoroughly your whole manuscript, I can understand that you invoke molecular docking method for ensuring the formation of protein-peptide complex. However, it is not elicited in your methodology section. Please kindly create a specific 'molecular docking' subsection there, so the reader can have better insight on your invoked methods.	Thank you for your suggestion. We used the template-based docking that utilizes homology modeling to build the peptide-MHC complexes [10–12]. The method’s subtitle had been changed from “Structural model building of T-cells epitope peptides and MHC complex” to “Template-based docking using homology modeling” (line number 118). The explanation as to why we prefer to use this method rather than the automatics peptide-protein docking program has been provided in the Discussion section (line number 417-426):<br><br>“*Before CGMD simulation, we prepared the initial conformation of peptide-MHC complex using protein-peptide docking. However, we identified at least three major challenges to protein-peptide docking: (i) modeling significant conformational changes of both peptide and protein molecules (flexibility problem); (ii) selection of the highest accuracy structure out of many generated models (scoring problem); and (iii) integration of experimental data and computational predictions into the protein–peptide docking scheme (integrative modeling). Interaction details between peptide epitopes and MHC-I molecules have been known, where the peptides anchor to the MHC-I binding pockets through their second and last residues. Such knowledge benefits us from employing the template-based docking method that utilizes homology modeling to prepare peptide-MHC (pMHC) complexes studied in this work. A similar approach has also been adopted by PANDORA, a technique to model pHMC with a success rate of 93%.”*

References

1. Liu J, Chandrashekar A, Sellers D, Barrett J, Jacob-Dolan C, Lifton M, et al. Vaccines elicit highly conserved cellular immunity to SARS-CoV-2 Omicron. Nature. 2022 Mar 17;603(7901):493–6.

2. Polack FP, Thomas SJ, Kitchin N, Absalon J, Gurtman A, Lockhart S, et al. Safety and Efficacy of the BNT162b2 mRNA Covid-19 Vaccine. New England Journal of Medicine. 2020 Dec 31;383(27):2603–15.

3. Möbs C, Schmidt T. Research Techniques Made Simple: Monitoring of T-Cell Subsets using the ELISPOT Assay. Vol. 136, Journal of Investigative Dermatology. Elsevier B.V.; 2016. p. e55–9.

4. Blache U, Weiss R, Boldt A, Kapinsky M, Blaudszun AR, Quaiser A, et al. Advanced Flow Cytometry Assays for Immune Monitoring of CAR-T Cell Applications. Front Immunol. 2021 May 3;12.

5. Anderson JL, Mota TM, Evans VA, Kumar N, Rezaei SD, Cheong K, et al. Understanding factors that modulate the establishment of HIV latency in resting CD4+ T-Cells in vitro. PLoS One. 2016 Jul 1;11(7).

6. Erawati M, Andriany M. Determinants of latent tuberculosis infection among nurses at public health centers in Indonesia. Original Research Belitung Nursing Journal. 2022;8(1):28–34.

7. Gilani B, Sergent SR. Interferon Test. In StatPearls Publishing; 2023.

8. Wyllie D, Jones HE, Mulchandani R, Trickey A, Taylor-Phillips S, Brooks T, et al. SARS-CoV-2 responsive T cell numbers and anti-Spike IgG levels are both associated with protection from COVID-19: A prospective cohort study in keyworkers. medRxiv. 2021;

9. Kruse M, Dark C, Aspden M, Cochrane D, Competiello R, Peltz M, et al. Performance of the T-SPOT ®.COVID test for detecting SARS-CoV-2-responsive T cells. International Journal of Infectious Diseases. 2021 Dec 1;113:155–61.

10. Logean A, Rognan D. Recovery of known T-cell epitopes by computational scanning of a viral genome. Vol. 16, Journal of Computer-Aided Molecular Design. 2002.

11. Kosmopoulou A, Vlassi M, Stavrakoudis A, Sakarellos C, Sakarellos-Daitsiotis M. T-cell epitopes of the La/SSB autoantigen: Prediction based on the homology modeling of HLA-DQ2/DQ7 with the insulin-B peptide/HLA-DQ8 complex. J Comput Chem. 2006 Jul 15;27(9):1033–44.

12. Khan JM, Ranganathan S. pDOCK: a new technique for rapid and accurate docking of peptide ligands to Major Histocompatibility Complexes. Immunome Res. 2010;6((Suppl 1):S2).
